# CEA clearance pattern as a predictor of tumor response to neoadjuvant treatment in rectal cancer: a post-hoc analysis of FOWARC trial

**DOI:** 10.1186/s12885-018-4997-y

**Published:** 2018-11-20

**Authors:** Huabin Hu, Jin Huang, Ping Lan, Lei Wang, Meijin Huang, Jianping Wang, Yanhong Deng

**Affiliations:** 1 Department of Medical Oncology, The Sixth Affiliated Hospital of Sun Yat-Sen University, Yuancunheng 2nd Road, Guangzhou, 510655 People’s Republic of China; 2 Department of Oncology, Xiangya Hospital of Central South University, Changsha, China; 3 Department of Colorectal Surgery, The Sixth Affiliated Hospital of Sun Yat-Sen University, Yuancunheng 2nd Road, Guangzhou, 510655 People’s Republic of China; 4 Guangdong Institute of Gastroenterology, Guangdong Provincial Key Laboratory of Colorectal and Pelvic Floor Diseases, Guangzhou, China

**Keywords:** Rectal cancer, Carcinoembryonic antigen, Neoadjuvant treatment, Pathologic complete response

## Abstract

**Background:**

The clinical factors that accurately predict the response to preoperative treatment in rectal cancer were yet unknown. The carcinoembryonic antigen (CEA) clearance pattern during neoadjuvant treatment has been developed and the predictive value explored in rectal cancer patients with elevated CEA levels (> 5 ng/mL).

**Methods:**

The training cohort was derived from the FOWARC prospective phase III trial, and 71/483 eligible patients were included. The validation cohort consisted of 75/587 consecutive rectal cancer patients from Xiangya Hospital between 2014 and 2015. The kinetic changes in serum CEA were measured at different time points during the neoadjuvant treatment. An exponential trend line was drawn using the CEA values. The patients were categorized into two groups based on the R^2^ value of the trend line, which indicates the correlation coefficient between the exponential graph and measured CEA values: exponential decrease group (0.9 < R^2^ ≤ 1.0) and non-exponential decrease group (R^2^ ≤ 0.9).

**Results:**

In multivariate analysis, the patients in the CEA exponential decrease group had significantly high adequate rate of downstaging (ypT0-2N0M0), and pathologic complete response (pCR) rates after neoadjuvant treatment in the training cohort. The predictive values of the CEA clearance pattern for tumor downstaging and pCR were further confirmed in an independent validation cohort.

**Conclusions:**

The CEA clearance pattern was an independent predictor of tumor response to neoadjuvant treatment in patients with rectal cancer. It might serve as an adjunct in the assessment of complete clinical response and guide individualized treatment strategies.

**Trial registration:**

NCT01211210.

**Electronic supplementary material:**

The online version of this article (10.1186/s12885-018-4997-y) contains supplementary material, which is available to authorized users.

## Background

Neoadjuvant chemoradiation therapy followed by total mesorectal excision (TME) for locally advanced rectal cancer has been considered as the optimal management strategy owing to increased control of local disease, decreased toxicity, and greater sphincter preservation rates [1–4]. Nevertheless, great differences in treatment response are continually observed among treated patients. Tumor downstaging can be obtained only in half of the cases, and a pathologic complete response (pCR) is 10–30% [5, 6].

An optimal knowledge of the factors that predict response to preoperative treatment may eventually lead to the development of individualized, risk-adapted treatment strategies for rectal cancer patients. Biochemical markers and clinical characteristics including serum carcinoembryonic antigen (CEA), tumor length, and circumferential tumor extent have been investigated as predictors [7–12].

CEA is a cell surface-anchored glycoprotein that was first identified in human colon cancer tissue extracts [13]. The levels CEA in pre- and post-treatment were described as independent predictive factors of response to neoadjuvant treatment [9, 14]. Other studies showed that the normalization of CEA levels after neoadjuvant treatment was correlated with pCR [15, 16]. The data on the evaluation of the dynamic changes in CEA levels suggested that a predictive indicator during neoadjuvant treatment of rectal cancer was scarce. Thus, the present study aimed to investigate the pattern of serum CEA clearance as a predictive tool for tumor response in rectal cancer patients receiving neoadjuvant treatment.

## Methods

### Patient population

The training cohort was derived from the FOWARC randomized prospective phase III trial exploring mFOLFOX6- and fluorouracil-based preoperative chemoradiotherapy in the locally advanced rectal cancer [17]. The patients were eligible for the present investigation if they fulfilled the following criteria: 1) received neoadjuvant chemoradiation followed by TME resection, 2) baseline CEA level was elevated (≥5 ng/mL), and 3) follow-up CEA values (2nd, 4th, 6th, 8th and 14th week from the beginning of the preoperative treatment) were available during the study. Finally, 71 patients were included in the training cohort (Additional file 1: Figure S1). The validation cohort consisted of 75/587 consecutive rectal cancer patients undergoing fluorouracil-based neoadjuvant chemoradiation and TME resection between 2014 and 2015 at the Xiangya Hospital of Central South University. The inclusion and exclusion criteria were similar to that of the FOWARC trial and those previously described in the training cohort, except that CEA was measured at four time points, including the baseline, 2nd, 6th, and 12th weeks from the start of the preoperative treatment (Additional file 2: Figure S2).

### Treatment

In the training cohort, patients in the fluorouracil-radiotherapy group received preoperative treatment with five cycles of infusional fluorouracil [leucovorin 400 mg/m^2^ intravenously, followed by fluorouracil 400 mg/m^2^, and fluorouracil 2.4 g/m^2^ by 48 h continuous intravenous infusion (de Gramont regimen)] with concurrent radiotherapy during cycles 2–4. The mFOLFOX6-radiotherapy group was administered similar treatment as the fluorouracil-radiotherapy group plus oxaliplatin 85 mg/m^2^ intravenously on day 1 of each chemotherapy cycle. Radiotherapy was administered at 1.8–2.0 Gy for 23–28 fractions over 5–6 weeks and a total dose of 46.0–50.4 Gy [17]. In the validation cohort, patients underwent 5 weeks of radiotherapy treatment at 50 Gy/25 fractions with concurrent capecitabine 800 mg/m^2^ two times daily for 5 consecutive days/week.

### Pathological assessment

All resection specimens were examined according to a standardized protocol that included TNM classification according to the American Joint Committee on the Cancer-International Union Against Cancer (seventh edition). pCR was defined as the absence of viable cells in the primary tumor and lymph nodes (ypT0N0) as evaluated by two pathologists blinded to the treatment group. Tumor regression grade (TRG) was performed semi-quantitatively by determining the amount of viable tumor versus fibrotic tissue that ranged from the lack of tumor regression to complete response with no viable tumor identified. The four groups of TRG classification were as follows: grade 0, total regression (no viable tumor cells; fibrotic mass only); grade 1, good regression (dominant fibrosis outgrowing tumor mass [ie, more than 50% tumor regression]); grade 2, moderate regression (dominant tumor mass with obvious fibrosis in 26 to 50% of tumor mass); grade 3, no regression or minor regression (dominant tumor mass with obvious fibrosis in 25% or less of the tumor mass) [18]. In the present study, good regression was defined as TRG 0–1, and good tumor downstaging was defined as ypT0-2N0M0 (ypStage0–1).

### CEA analysis and clearance

All of the serum CEA assays were performed within one laboratory by the Elecsys 2010 electrochemiluminescence immunoassay (Roche Diagnostics) in which the reference normal range was 0-5 ng/ml. The serum concentration of CEA was measured at the baseline (within 7 days before preoperative treatment start) and then during the preoperative treatment (training cohort: at 2nd, 4th, 6th, 8th, and 14th weeks from the start of preoperative treatment; validation cohort: at the 2nd, 6th, and 12th weeks from the start of preoperative treatment).

Our initial hypothesis postulated that the CEA decay and production, rendered an exponential distribution depending on the state of tumor activity. If the tumor is more sensitive to the neoadjuvant chemoradiation, the CEA produced by the rectal cancer tissue is suppressed more thoroughly. As a result, the clearance pattern of CEA follows the classical the exponential kinetics format [19–22].

**Fig. 1:**
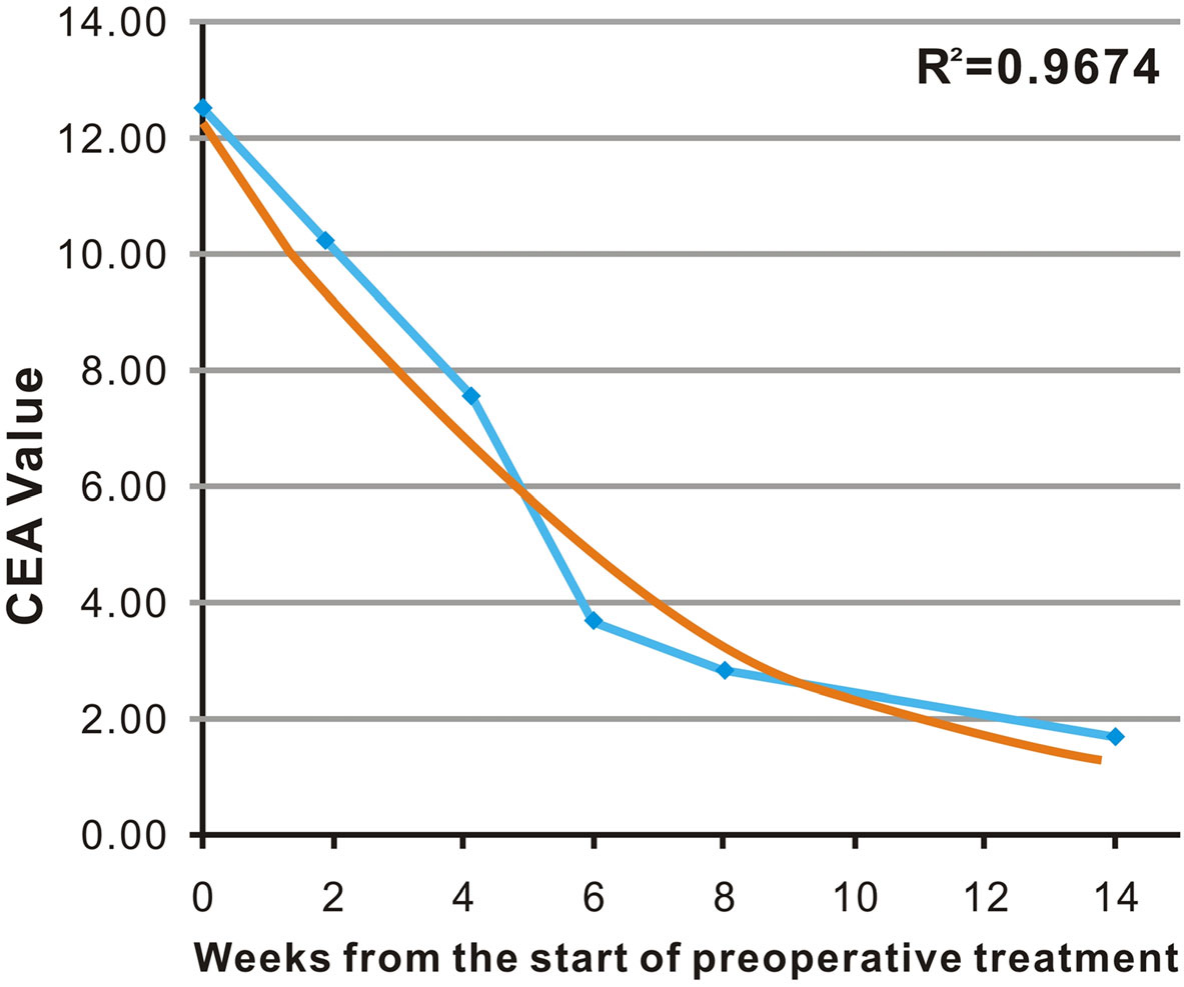
An exponential trend line (blue line) was drawn using each CEA value (baseline, 2nd, 4th, 6th, 8th, and 14th week from the start of preoperative treatment). The function of the exponential curve (yellow line) was drawn. R^2^ values were calculated as the deviation between the calculated curves and the measured CEA value

The exponential curves were drawn based on the trend line, and R^2^ values calculated. R^2^ indicates the correlation coefficient between the trend line illustrating the exponential decrease and the measured CEA values. If R^2^ = 1, all the data points will fall on the exponential line. Therefore, the closer the R^2^ value to 1, more CEA values fit the exponential curve, and the patients with exponential CEA clearance potentially displayed an adequate tumor response (Fig. 1). We categorized the patients into two groups based on the R^2^ values: exponential decrease group (0.9 < R^2^ ≤ 1.0), and non-exponential decrease group (R^2^ ≤ 0.9).

### Statistical analysis

Comparison of variables was performed with the Χ2 test or Fisher exact test for qualitative variables, and the Student t test was used for quantitative variables. Univariate and multivariate analyses were performed by using the Student t test and logistic regression to identify predictors for the end points TRG, tumor downstaging and pCR. Only factors for which *p* ≤ 0.05, as determined in the univariate analysis in the training cohort, were entered into the multivariate analysis in the training and validation cohort. The area under the curve (AUC) is used as a summary measure of the receiver operating characteristics (ROC) curve (which plots sensitivity versus 1-specificity) and represents the discrimination ability. AUC is expressed on a scale of 0.5 to 1. The larger the AUC value, the better the classification effect. In this study, AUC represents the prediction accuracy of CEA clearance pattern and clinical parameters for TRG, tumor downstaging, and pCR. To determine whether the CEA was in the normal range, 5 ng/mL was used as the cutoff. For all analyses, P < < 0.05 was considered statistically significant. Statistical analyses were performed using software SPSS versions 22 (SPSS Inc. Chicago, IL, USA) and MedCalc version 13.0 (MedCalcSoftware).

## Results

### Patient characteristics

The clinicopathological characteristics of patients are shown in Table 1. In the training cohort, 32 patients (45%) comprised the fluorouracil-radiotherapy group, and 39 (55%) formed the mFOLFOX6-radiotherapy group. The mean pre- or post-chemoradiation CEA level was 27 and 5 ng/mL, respectively. The CEA level in 47 patients (66%) dropped to the normal range (≤5 ng/mL) after 6–8 weeks from the completion of chemoradiation.

**Table 1:** Clinicopathological characteristics of the training (*n* = 71) and validation cohorts (*n* = 75)

Clinicopathologic indexes	Training cohort (*n* = 71)	Validation cohort (*n* = 75)	P
Age (mean, y ± SD)	54 ± 12	54 ± 12	0.801
Gender			**0.011**
Male	47 (66%)	34 (45%)	
Female	24 (34%)	41 (55%)	
Tumor differentiation			**0.005**
Well/Moderately	34 (48%)	56 (75%)	
Poorly/Undifferentiated	37 (52%)	19 (25%)	
Distance from anal verge			0.259
≦5 cm	35 (49%)	30 (40%)	
> 5 cm	36 (51%)	45 (60%)	
Tumor length (median; range)	4 (2–12)	4 (2–11)	0.948
Circumferential extent			0.113
≦50%	17 (24%)	27 (36%)	
> 50%	54 (76%)	48 (64%)	
Pretreatment T stage			**< 0.001**
T_2_	0	6 (8%)	
T_3_	52 (73%)	62 (83%)	
T_4_	19 (27%)	7 (9%)	
Pretreatment N stage			0.119
N_0_	12 (17%)	21 (28%)	
N_1_	30 (42%)	34 (45%)	
N_2_	29 (41%)	20 (27%)	
Pre- treatment CEA level (mean, ng/mL; rang)	27 (5–200)	14 (5–57)	**0.001**
Post- treatment CEA level (mean, ng/mL; rang)	5 (0.5–45)	6 (0.5–34)	0.605
Normalization of post- treatment CEA Level			0.541
Normal (< 5 ng/mL)	47 (66%)	46 (61%)	
Elevated (≧5 ng/mL)	24 (34%)	29 (39%)	
CEA clearance pattern			0.599
Exponential decrease (R^2^≧0.9)	31 (44%)	36 (48%)	
Non-exponential decrease (R^2^ < 0.9)	40 (56%)	39 (52%)	

The *p* value in boldface means statistically significant, that is, less than 0.05

In the validation cohort, all patients underwent a neoadjuvant radiotherapy with concurrent capecitabine. The mean pre-chemoradiation CEA level was 14 ng/mL, which was significantly lower as compared to the training cohort. The mean post-chemoradiation CEA level was 6 ng/mL, and the normalization of CEA rate was 61% (46 patients).

44% (31/71) and 48% (36/75) patients were categorized into the CEA exponential decrease group in the training and the validation cohorts, respectively. The clinicopathological characteristics were similar between the two cohorts, except gender, tumor differentiation, and pre-treatment T stage. The validation cohort included fewer males (45 vs. 66%), and patients exhibited poor tumor differentiation (25 vs. 52%), and T4 (9 vs. 27%) than those in the validation cohort (Table 1).

### Association of CEA clearance pattern with clinicopathological characteristics

In the training cohort, we found that the patients in the CEA exponential decrease group were associated with circumferential extent ≤50% (*P* = 0.001); whereas, the relationship between the CEA clearance pattern and clinicopathological parameters for the validation cohort was not statistically significant (Additional file 3: Table S1).

### Predictive significance of CEA clearance pattern in the training cohort

In the training cohort, 33 patients (46%) showed TRG 0–1, 20 patients (28%) exhibited good downstaging, and 11 patients (15%) had pCR. The results from our univariate analysis indicated that the tumor length was ≤4 cm (*P* = 0.015), circumferential extent ≤50% (*P* = 0.022), normalization of post-treatment CEA level <  5 ng/mL (*P* = 0.037), and CEA exponential decrease (*P* = 0.028) were associated with good TRG (0–1) (Table 2). The pre-treatment T stage (T3 vs. T4) (*P* = 0.009), well/moderate tumor differentiation (*P* = 0.02), and CEA exponential decrease (*P* = 0.001) were associated with a high adequate rate of downstaging (Table 3). The patients with tumor length ≤ 4 cm (*P* = 0.044) or CEA exponential decrease (*P* = 0.008) potentially achieved a pCR (Table 4). Other clinical factors such as age, gender, pre-treatment N stage, distance from the anal verge, pre- or post-treatment CEA level, and concurrent chemotherapy regimen exerted no predictive significance for TRG, tumor downstaging, and pCR.

**Table 2:** Univariate analysis of predictors for TRG in the training and validation cohorts

Variable	Training cohort			Validation cohort		
	TRG 0–1	TRG 2–3	*P*	TRG 0–1	TRG 2–3	*P*
No. of patients	33	38		33	42	
Age (mean, y ± SD)	52 ± 12	56 ± 12	0.199	55 ± 13	55 ± 11	0.827
Gender			0.671			0.627
Male	21 (64%)	26 (68%)		16 (49%)	18 (43%)	
Female	12 (36%)	12 (32%)		17 (51%)	24 (57%)	
Pretreatment T stage			0.655			0.854
T_2_	0	0		2 (6%)	4 (9%)	
T_3_	25 (76%)	27 (71%)		28 (85%)	34 (82%)	
T_4_	8 (24%)	11 (29%		3 (9%)	4 (9%)	
Pretreatment N stage			0.928			0.638
N_0_	5 (16%)	7 (18%)		10 (30%)	11 (26%)	
N_1_	14 (42%)	16 (42%)		16 (49%)	18 (43%)	
N_2_	14 (42%)	15 (40%)		7 (21%)	13 (31%)	
Tumor differentiation			0.128			0.732
Well/Moderately	19 (58%)	15 (40%)		24 (73%)	32 (76%)	
Poorly/Undifferentiated	14 (42%)	23 (60%)		9 (27%)	10 (24%)	
Distance from the anal verge			0.120			0.924
≦5 cm	13 (39%)	22 (58%)		13 (39%)	17 (41%)	
> 5 cm	20 (61%)	16 (42%)		20 (61%)	25 (59%)	
Tumor length			**0.015**			**0.011**
≦4 cm	19 (42%)	11 (71%)		19 (58%)	12 (29%)	
> 4 cm	14 (58%)	27 (29%)		14 (42%)	30 (64%)	
Circumferential extent			**0.022**			0.163
≦50%	12 (36%)	5 (13%)		9 (27%)	18 (43%)	
> 50%	21 (64%)	33 (87%)		24 (73%)	24 (57%)	
Pre-treatment CEA level (mean, ng/ml; 95%CI)	29 (16–43)	25 (17–34)	0.615	15 (11–18)	14 (11–16)	0.548
Post- treatment CEA level (mean, ng/mL; 95%CI)	4 (1–7)	6 (4–9)	0.233	5 (4–7)	7 (4–9)	0.343
Normalization of post- treatment CEA Level			**0.037**			0.187
Normal (< 5 ng/mL)	26 (79%)	21 (55%)		23 (70%)	23 (55%)	
Elevated (≧5 ng/mL)	7 (21%)	17 (45%)		10 (30%)	19 (45%)	
CEA clearance pattern			**0.028**			**0.016**
Exponential decrease (R^2^≧0.9)	19 (58%)	12 (32%)		21 (64%)	15 (36%)	
Non-exponential decrease (R^2^ < 0.9)	14 (42%)	26 (68%)		12 (36%)	27 (64%)	
Concurrent chemotherapy			0.135			NA
Capecitabine	0	0		33 (44%)	42 (56%)	
Infusional fluorouracil	15 (45%)	24 (63%)		0	0	
mFOLFOX6	48 (55%)	14 (37%)		0	0	

Abbreviation: *NA* not applicable, *TRG* tumor regression grade

The *p* value in boldface means statistically significant, that is, less than 0.05

**Table 3:** Univariate analysis of predictors for tumor downstaging in the training and validation cohorts

Variable	Training cohort			Validation cohort		
	Good downstaging	Poor downstaging	*P*	Good downstaging	Poor downstaging	*P*
No. of patients	20	51		26	49	
Age (mean, y ± SD)	58 ± 10	52 ± 13	0.101	54 ± 10	55 ± 13	0.630
Gender			0.671			0.701
Male	14 (70%)	33 (65%)		11 (42%)	23 (47%)	
Female	6 (30%)	18 (35%)		15 (58%)	26 (53%)	
Pretreatment T stage			**0.009**			0.125
T_2_	0	0		2 (8%)	4 (8%)	
T_3_	19 (95%)	33 (65%)		24 (92%)	38 (78%)	
T_4_	1 (5%)	18 (35)		0	7 (14%)	
Pretreatment N stage			0.160			0.858
N_0_	2 (10%)	10 (20%)		8 (31%)	13 (26%)	
N_1_	12 (60%)	18 (35%)		12 (46%)	22 (45%)	
N_2_	6 (30%))	23 (45%)		6 (23%)	14 (29%)	
Tumor differentiation			**0.020**			0.818
Well/Moderately	14 (70%)	20 (40%)		19 (73%)	37 (76%)	
Poorly/Undifferentiated	6 (30%)	31 (60%)		7 (27%)	12 (24%)	
Distance from the anal verge			0.327			0.235
≦5 cm	8 (40%)	27 (53%)		8 (31%)	22 (45%)	
> 5 cm	12 (60%)	24 (47)		18 (69%)	27 (55%)	
Tumor length			0.058			0.267
≦4 cm	12 (60%)	18 (65%)		13 (50%)	18 (37%)	
> 4 cm	8 (40%)	33 (35%)		13 (50%)	31 (63%)	
Circumferential extent			0.065			0.856
≦50%	8 (40%)	9 (18%)		9 (35%)	18 (37%)	
> 50%	12 (60%)	42 (82%)		17 (65%)	31 (63%)	
Pre- treatment CEA level (mean, ng/mL; 95%CI)	23 (14–33)	28 (19–39)	0.551	17 (12–22)	13 (11–15)	0.066
Post- treatment CEA level (mean, ng/mL; 95%CI)	5 (0.2–9)	6 (4–8)	0.680	6 (4–9)	6 (4–7)	0.712
Normalization of post- treatment CEA Level			0.124			0.600
Normal (< 5 ng/mL)	16 (80%)	31 (61%)		17 (65%)	29 (60%)	
Elevated (≧ 5 ng/mL)	4 (20%)	20 (39%)		9 (35%)	20 (40%)	
CEA clearance pattern			**0.001**			**0.007**
Exponential decrease (R^2^≧0.9)	15 (75%)	16 (31%)		18 (69%)	18 (37%)	
Non-exponential decrease (R^2^ < 0.9)	5 (25%)	35 (69%)		8 (31%)	31 (63%)	
Concurrent chemotherapy			0.601			NA
Capecitabine	0	0		26 (35%)	49 (65%)	
Infusional fluorouracil	10 (50%)	29 (57%)		0	0	
mFOLFOX6	10 (50%)	22 (43%)		0	0	

Abbreviation: *NA* not applicable

The *p* value in boldface means statistically significant, that is, less than 0.05

**Table 4:** Univariate analysis of predictors for pCR in the training and validation cohorts

Variable	Training cohort			Validation cohort		
	pCR	No pCR	*P*	pCR	No pCR	*P*
No. of patients	11	60		10	65	
Age (mean, y ± SD)	57 ± 12	54 ± 13	0.323	55 ± 13	55 ± 12	0.910
Gender			0.618			0.497
Male	8 (73%)	39 (65%)		6 (60%)	28 (43%)	
Female	3 (27%)	21 (35%)		4 (40%)	37 (57%)	
Pretreatment T stage			0.267			0.546
T_2_	0	0		1 (10%)	5 (8%)	
T_3_	10 (9%)	42 (70%)		9 (90%)	53 (81%)	
T_4_	1 (91%)	18 (30%)		0	7 (11%)	
Pretreatment N stage			0.217			0.121
N_0_	2 (18%)	10 (17%)		4 (40%)	17 (26%)	
N_1_	7 (64%)	23 (38%)		6 (60%)	28 (43%)	
N_2_	2 (18%)	27 (45%)		0	20 (31%)	
Tumor differentiation			0.073			0.707
Well/Moderately	8 (73%)	26 (43%)		7 (70%)	49 (75%)	
Poorly/Undifferentiated	3 (27%)	34 (57%)		3 (30%)	16 (25%)	
Distance from the anal verge			0.301			0.731
≦5 cm	7 (64%)	28 (53%)		3 (30%)	27 (42%)	
> 5 cm	4 (36%)	32 (47%)		7 (70%)	38 (58%)	
Tumor length			**0.044**			0.509
≦4 cm	8 (73%)	22 (37%)		3 (30%)	28 (43%)	
> 4 cm	3 (27%)	38 (63%)		7 (70%)	37 (57%)	
Circumferential extent			0.441			0.480
≦50%	4 (36%)	13 (22%)		5 (50%)	22 (34%)	
> 50%	7 (64%)	47 (78%)		5 (50%)	43 (66%)	
Pre-treatment CEA level (mean, ng/mL; 95%CI)	24 (10–38)	28 (19–37)	0.709	13 (11–15)	20 (10–30)	0.165
Post- treatment CEA level (mean, ng/mL; 95%CI)	2 (0.2–4)	6 (4–8)	0.164	6 (2–11)	6 (5–7)	0.808
Normalization of post- treatment CEA Level			0.312			1.000
Normal (< 5 ng/mL)	9 (82%)	38 (63%)		6 (60%)	40 (62%)	
Elevated (≧5 ng/mL)	2 (18%)	22 (37%)		4 (40%)	25 (38%)	
CEA clearance pattern			**0.008**			**0.042**
Exponential decrease (R^2^≧0.9)	9 (82%)	22 (37%)		8 (80%)	28 (43%)	
Non-exponential decrease (R^2^ < 0.9)	2 (18%)	38 (63%)		2 (20%)	37 (57%)	
Concurrent chemotherapy			0.743			NA
Capecitabine	0	0		10 (13%)	65 (87%)	
Infusional fluorouracil	7 (64%)	32 (47%)		0	0	
mFOLFOX6	4 (36%)	28 (53%)		0	0	

Abbreviation: *NA* not applicable, *pCR* pathologic complete response

The *p* value in boldface means statistically significant, that is, less than 0.05

**Fig. 2:**
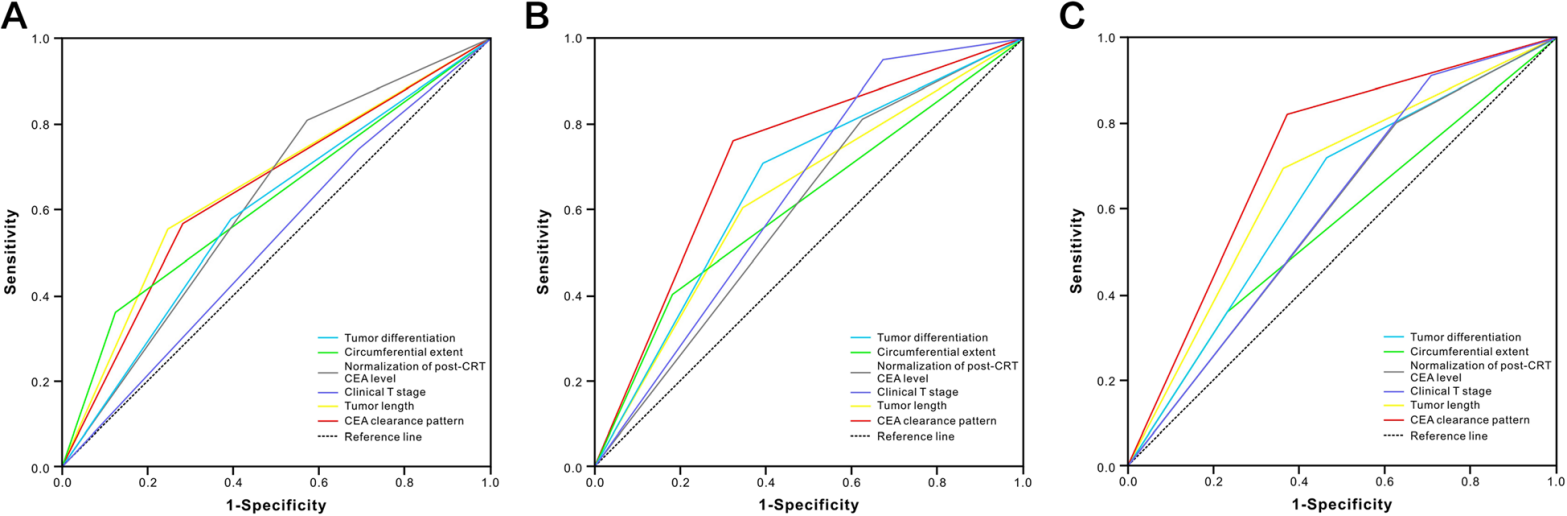
Predictive ability of the CEA clearance pattern for TRG (**a**), tumor downstaging (**b**), and pCR (**c**) were compared to the other clinical parameters by AUC curves in the training cohort

According to the multivariate analysis, the CEA clearance pattern was independent predictive factor for tumor downstaging [Odds ratio (OR), 8.25; 95% CI 2.19–31.10; *P* = 0.002; Table 5) and pCR (OR, 8.30; 95% CI, 1.56–44.17; *P* = 0.013) (Table 6). The AUC for the CEA clearance pattern was 0.63 (95% CI, 0.50–0.76), 0.72 (95% CI, 0.59–0.85), and 0.73 (95% CI, 0.57–0.88), respectively (Fig. 2).

**Table 5:** Multivariate regression analysis with tumor downstaging as dependent variable

Variable	Good downstaging	Poor downstaging	*P*	Odds Ratio (95%CI)
Training cohort				
Pretreatment T stage			**0.04**	10.14 (1.11–92.55)
T2–3	19 (95%)	33 (65%)		
T4	1 (5%)	18 (35)		
Tumor differentiation			**0.032**	4.21 (1.13–15.68)
Well/Moderately	14 (70%)	20 (40%)		
Poorly/Undifferentiated	6 (30%)	31 (60%)		
CEA clearance pattern			**0.002**	8.25 (2.19–31.10)
Exponential decrease (R^2^≧0.9)	15 (75%)	16 (31%)		
Non-exponential decrease (R^2^ < 0.9)	5 (25%)	35 (69%)		
Validation cohort				
Pretreatment T stage			0.108	0.31 (0.08–1.29)
T2–3	26 (78%)	42 (86%)		
T4	0	7 (14%)		
Tumor differentiation			0.752	1.22 (0.36–4.09)
Well/Moderately	19 (73%)	37 (76%)		
Poorly/Undifferentiated	7 (27%)	12 (24%)		
CEA clearance pattern			**0.008**	4.28 (1.47–12.43)
Exponential decrease (R^2^≧0.9)	18 (69%)	18 (37%)		
Non-exponential decrease (R^2^ < 0.9)	8 (31%)	31 (63%)		

The *p* value in boldface means statistically significant, that is, less than 0.05

**Table 6:** Multivariate regression analysis with pCR as dependent variable

Variable	pCR	No pCR	*P*	Odds Ratio (95%CI)
Training cohort				
Tumor length			**0.037**	4.99 (1.10–22.63)
≦4 cm	8 (73%)	22 (37%)		
> 4 cm	3 (27%)	38 (63%)		
CEA clearance pattern			**0.013**	8.30 (1.56–44.17)
Exponential decrease (R^2^≧0.9)	9 (82%)	22 (37%)		
Non-exponential decrease (R^2^ < 0.9)	2 (18%)	38 (63%)		
Validation cohort				
Tumor length			0.484	0.59 (0.13–2.59)
≦4 cm	3 (30%)	28 (43%)		
> 4 cm	7 (70%)	37 (57%)		
CEA clearance pattern			**0.047**	5.22 (1.02–26.60)
Exponential decrease (R^2^≧0.9)	8 (80%)	28 (43%)		
Non-exponential decrease (R^2^ < 0.9)	2 (20%)	37 (57%)		

Abbreviation: *pCR* pathologic complete response

The *p* value in boldface means statistically significant, that is, less than 0.05

### Validation of CEA clearance pattern in an independent cohort

Forty four patients (44%) exhibited TRG 0–1, and 26 (35%) had good downstaging. A pCR was observed in 10 patients (13%). The CEA clearance pattern served as a predictor of tumor response to neoadjuvant treatment, which was further confirmed in an independent validation cohort of 75 patients. These results were similar to those obtained from the training cohort (Tables 2, 3, 4). The exponential decrease of CEA was associated with TRG 0–1 (*P* = 0.016), good downstaging (*P* = 0.007), and pCR (*P* = 0.042).

**Fig. 3:**
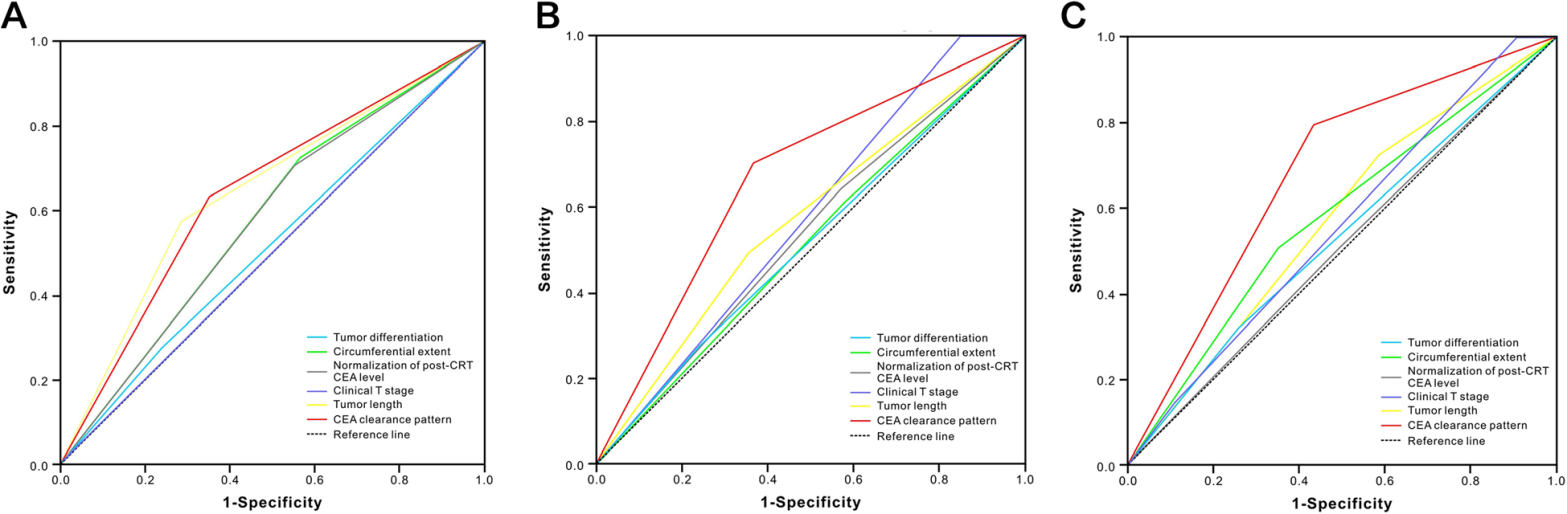
Predictive ability of the CEA clearance pattern for TRG (**a**), tumor downstaging (**b**), and pCR (**c**) were compared to the other clinical parameters by AUC curves in the validation cohort

In addition, the multivariate analysis considered the clinical parameters same as those in the training cohort. The CEA clearance pattern remained a significant predictive factor for TRG (OR, 4.37; 95% CI, 1.44–13.23; *P* = 0.009; Table 7), tumor downstaging (OR, 4.28; 95% CI, 1.47–12.43; *P* = 0.008; Table 5), and pCR (OR, 5.22; 95% CI, 1.02–26.60; *P* = 0.047; Table 6). The ability of discrimination of the CEA clearance pattern, as assessed by AUC, was 0.64 (95% CI, 0.51–0.77), 0.66 (95% CI, 0.53–0.79), and 0.69 (95% CI, 0.52–0.85) for TRG, tumor downstaging, and pCR (Fig. 3), respectively.

**Table 7:** Multivariate regression analysis with TRG as dependent variable

Variable	TRG 0–1	TRG 2–3	*P*	Odds Ratio (95%CI)
Training cohort				
Tumor length			**0.037**	3.19 (1.07–9.46)
≦4 cm	19 (42%)	11 (71%)		
> 4 cm	14 (58%)	27 (29%)		
Circumferential extent			0.146	2.65 (0.71–9.86)
≦50%	12 (36%)	5 (13%)		
> 50%	21 (64%)	33 (87%)		
Normalization of post- treatment CEA Level			0.082	2.81 (0.88–9.01)
Normal (< 5 ng/mL)	26 (79%)	21 (55%)		
Elevated (≧5 ng/mL)	7 (21%)	17 (45%)		
CEA clearance pattern			0.161	2.18 (0.73–6.44)
Exponential decrease (R^2^≧0.9)	19 (58%)	12 (32%)		
Non-exponential decrease (R^2^ < 0.9)	14 (42%)	26 (68%)		
Validation cohort				
Tumor length			**0.009**	4.23 (1.43–12.50)
≦4 cm	19 (58%)	12 (29%)		
> 4 cm	14 (42%)	30 (64%)		
Circumferential extent			0.147	0.42 (0.13–1.35)
≦50%	9 (27%)	18 (43%)		
> 50%	24 (73%)	24 (57%)		
Normalization of post- treatment CEA Level			0.884	0.92 (0.29–2.92)
Normal (< 5 ng/mL)	23 (70%)	23 (55%)		
Elevated (≧5 ng/mL)	10 (30%)	19 (45%)		
CEA clearance pattern			**0.009**	4.37 (1.44–13.23)
Exponential decrease (R^2^≧0.9)	21 (64%)	15 (36%)		
Non-exponential decrease (R^2^ < 0.9)	12 (36%)	27 (64%)		

Abbreviation: *TRG* tumor regression grade

The *p* value in boldface means statistically significant, that is, less than 0.05

## Discussion

CEA is the most widely used and readily available tumor marker for the management of colorectal cancer. The diagnostic and prognostic value of this marker has been extensively evaluated [23–27]. Recent studies have investigated its potential predictive value in tumor response to neoadjuvant treatment of rectal cancer. Wallin et al. recruited 530 patients and found that low pre-treatment CEA (3.4 vs. 9.6 ng/mL) was significantly associated with pCR in the multivariate analysis. When stratifying for smoking status, the predictive value was significant only in the nonsmokers [28]. Huh et al. demonstrated that low pre-treatment CEA level (< 5 ng/mL), non-circumferentiality, and non-macroscopic ulceration comprised of the independent predictors of a high pCR rate [9]. Yang et al. showed that low post-treatment CEA levels could predict of pCR. Especially, using ROC curves, the study determined that 2.61 ng/mL was the optimal cut-off level for CEA with a sensitivity and specificity of 76 and 58.4%, respectively [16]. Perez et al. demonstrated that post-treatment CEA levels < 5 ng/mL were predictive of both pCR as well as 5-year overall survival [14]. Kleiman et al. demonstrated that the normalization of post-treatment CEA in patients with elevated pre-treatment CEA levels was a significant predictor of pCR resulting in an approximately 65-fold potential increase in achieving pCR [15].

Contrary to previous studies, we did not find that patients achieving an good TRG, satisfactory tumor downstaging, and pCR were significantly associated with pre- or post-treatment CEA levels and normalization of post-treatment CEA according to the multivariate analysis. Thus, we hypothesized that the pre-treatment CEA value only reflected the tumor burden up to a specific extent but not the efficacy of the neoadjuvant treatment. The post-treatment CEA values would be less capable of providing the tumor response to neoadjuvant treatment as compared to the dynamic changes in CEA levels as they represent only one time point. Moreover, Kim et al. demonstrated that the pattern of serum CEA clearance after radical tumor resection in rectal cancer patients could be used as a surrogate marker for predicting cancer-specific mortality [22].

To the best of our knowledge, this is the first study evaluating the dynamic changes in CEA levels during neoadjuvant treatment that serves as a predictor for rectal cancer patients in two independent cohorts. In the present study, the CEA clearance pattern was constructed and categorized based on the R^2^ value, which was calculated by drawing the exponential trend lines using serially estimated CEA; this method was similar to that of Kim et al. [22]. Furthermore, our data showed that the patients in the CEA exponential decrease group defined by R^2^ > 0.9 had significantly good TRG, good tumor downstaging, and pCR rates after neoadjuvant treatment as assessed in a cohort of patients in the FOWARC trial [17]. A similar predictive effect of CEA clearance pattern on tumor response to neoadjuvant treatment was validated in an independent consecutive retrospective cohort. The predictive ability of the both cohort was stronger than that of the pre- or post-treatment CEA levels, normalization of CEA levels, and other conventional variables such as pre-treatment T or N stage, tumor differentiation, distance from the anal verge, tumor length, and circumferential extent.

According to the response evaluation criteria in solid tumors (RECIST version 1.1), if markers are initially above the upper normal limit, one of the criteria for assessing clinical complete response for a patient is the reduction of tumor markers to normal levels. Our study demonstrated that the pattern of CEA clearance might substitute for normalization of CEA level and be used as an adjunct in the assessment of complete clinical response that can guide individualized treatment strategies such as “Watch & Wait” [29–31]. In addition, the measure of CEA clearance pattern was established according to the baseline and the follow-up CEA values during neoadjuvant treatment, which are normally available in the clinical practice.

Nevertheless, the limitations of our study are as follows: 1) In the both training and validation cohort, baseline and follow-up CEA data could be evaluated only for a partial study population and in a single institution; 2) In the training cohort, the exponential decrease of CEA was significantly associated with good TRG as assessed by univariate analysis; however, statistical significance was not observed by multivariate analysis. These inconsistent results could be attributed to small sample sizes and the quality of retrospective cohort; 3) CEA is known to be elevated in smokers, and therefore a different reference value for a positive test is used. When assessing the predictive value of CEA dynamic changes, the influence of the patient’s smoking status is unclear. There is a potential negative effect of smoking on the efficacy of neoadjuvant chemoradiotherapy, however, we did not take into account the possible interaction of CEA clearance pattern with smoking due to absence of data.

## Conclusion

Our results demonstrated the CEA clearance pattern, a low cost and reproducible clinical parameter, was an independent predictor of tumor response to neoadjuvant treatment in patients with rectal cancer. It might serve as an adjunct in the assessment of complete clinical response and guide individualized treatment strategies.

## Additional files

Additional file 1: **Figure S1.** Schematic representation of the eligible patients included in the training cohort. (JPG 133 kb)

Additional file 2: **Figure S2.** Schematic representation of the eligible patients included in the validation cohort. (JPG 137 kb)

Additional file 3: **Table S1**. Correlation between the CEA clearance pattern and clinicopathological parameters. (DOCX 22 kb)

## Abbreviations

AUC: The area under the curves
CEA: Carcinoembryonic antigen
OR: Odds ratio
pCR: Pathologic complete response
RECIST: Response evaluation criteria in solid tumors
TME: Total mesorectal excision;
TRG: Tumor regression grade
