# Supplementary figures and images for: CEA clearance pattern as a predictor of tumor response to neoadjuvant treatment in rectal cancer: a post-hoc analysis of FOWARC trial
Source: BMC Cancer. 2018 Nov 20;18:1145. doi: 10.1186/s12885-018-4997-y (PMC6247708; doi:10.1186/s12885-018-4997-y)

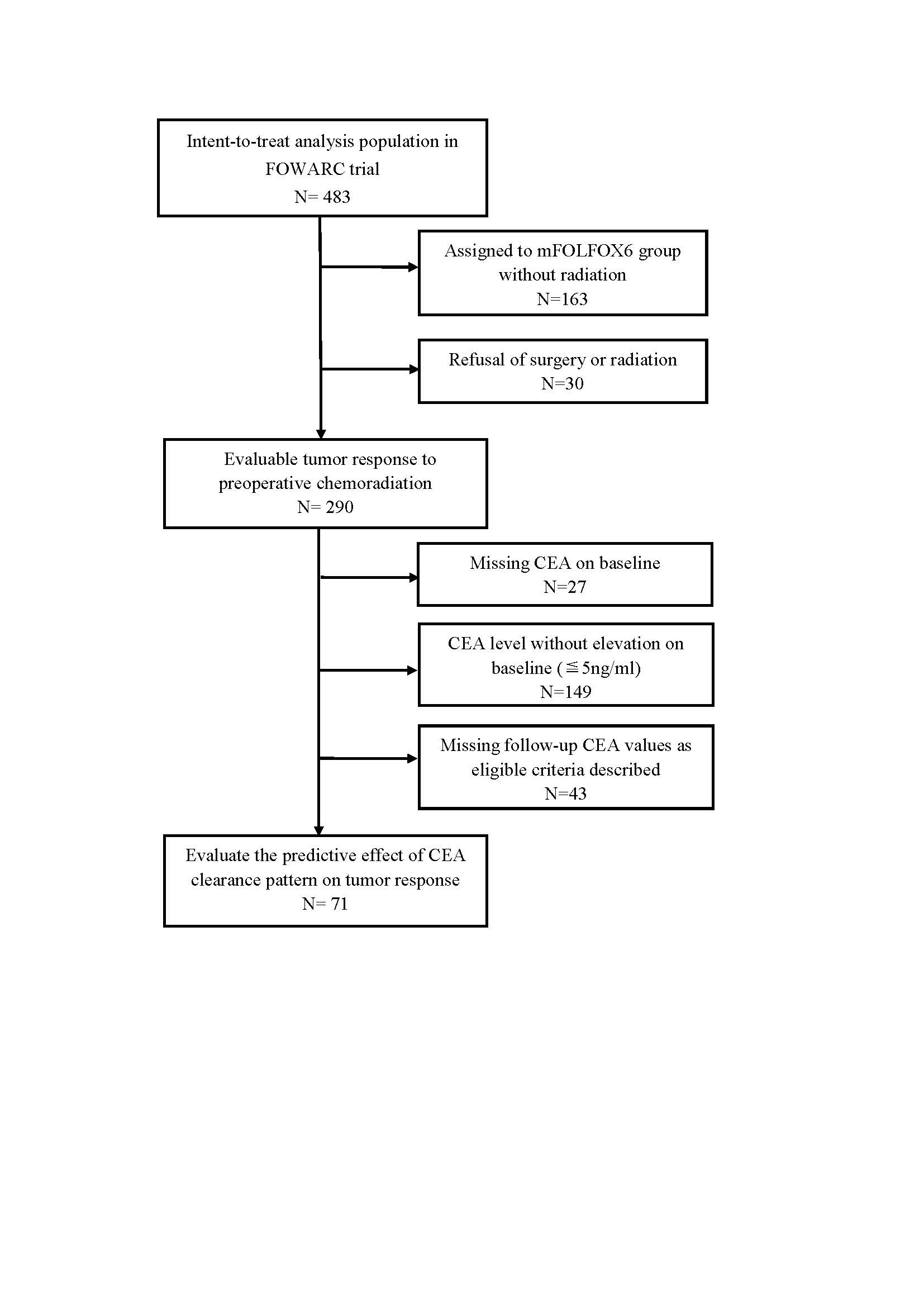

Supplement: Supplementary file 1 — Figure S1. Schematic representation of the eligible patients included in the training cohort. (JPG 133 kb) [file 12885_2018_4997_MOESM1_ESM.jpg]

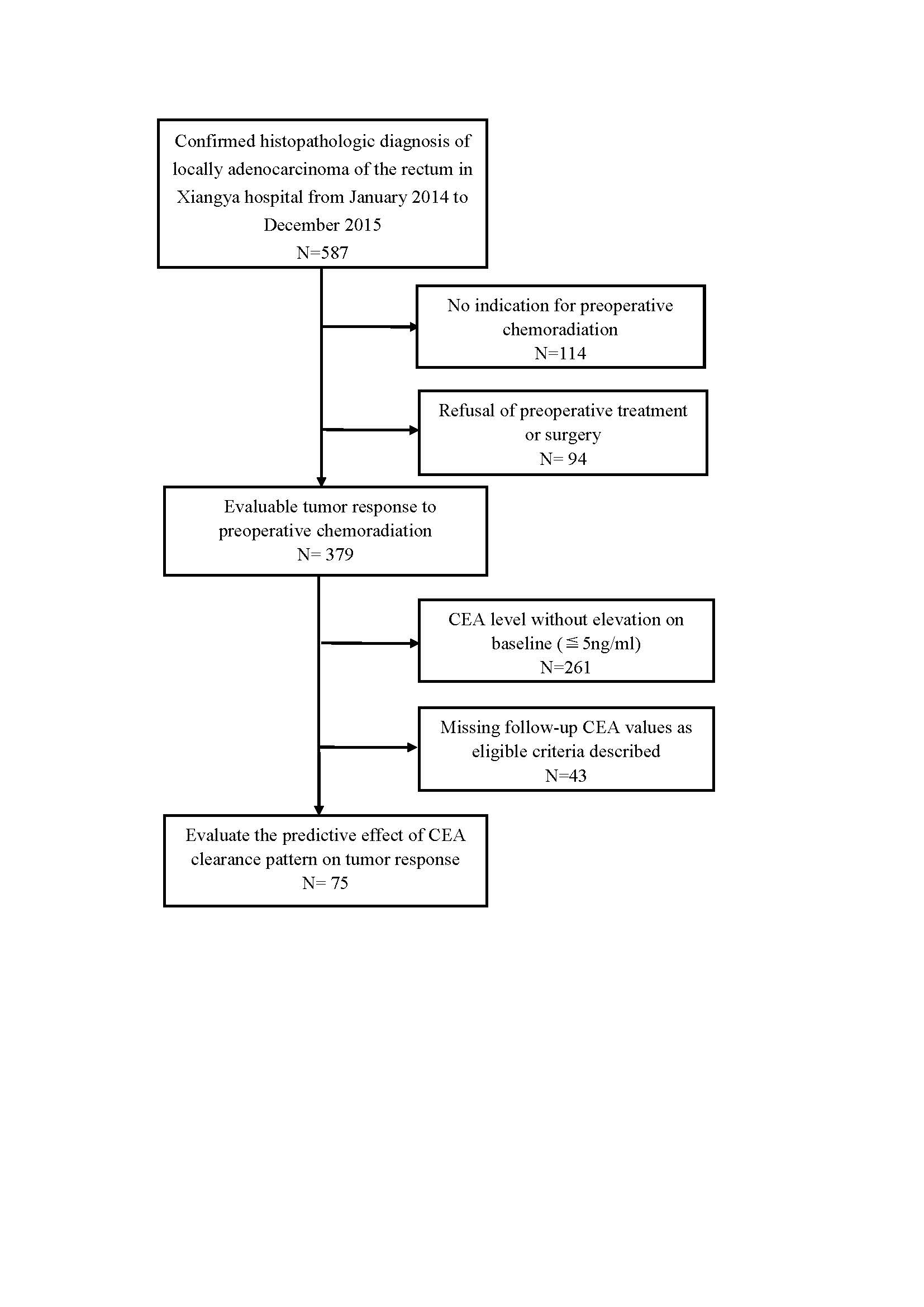

Supplement: Supplementary file 2 — Figure S2. Schematic representation of the eligible patients included in the validation cohort. (JPG 137 kb) [file 12885_2018_4997_MOESM2_ESM.jpg]
