# Supplementary material for: CEA clearance pattern as a predictor of tumor response to neoadjuvant treatment in rectal cancer: a post-hoc analysis of FOWARC trial
Source: BMC Cancer. 2018 Nov 20;18:1145. doi: 10.1186/s12885-018-4997-y (PMC6247708; doi:10.1186/s12885-018-4997-y)
Supplement: Supplementary file 3 — Table S1. Correlation between the CEA clearance pattern and clinicopathological parameters. (DOCX 22 kb) [file 12885_2018_4997_MOESM3_ESM.docx]

| **Variable** | **Training cohort** | | |  | **Validation cohort** | | |
| --- | --- | --- | --- | --- | --- | --- | --- |
|  | **Exponential decrease** | **Non-exponential decrease** | ***P*** |  | **Exponential decrease** | **Non-exponential decrease** | ***P*** |
| No. of patients | 31 | 40 |  |  | 36 | 39 |  |
| Age (mean, y ± SD) | 54 ± 10 | 55 ± 14 | 0.764 |  |  |  |  |
| Gender |  |  | 0.809 |  |  |  | 0.088 |
| Male | 21 | 26 |  |  | 20 | 14 |  |
| Female | 10 | 14 |  |  | 16 | 25 |  |
| Pretreatment T stage |  |  | 0.484 |  |  |  | 0.112 |
| T_2_ | 0 | 0 |  |  | 1 | 5 |  |
| T_3_ | 7 | 12 |  |  | 33 | 29 |  |
| T_4_ | 24 | 28 |  |  | 2 | 5 |  |
| Pretreatment N stage |  |  | 0.341 |  |  |  | 0.116 |
| N_0_ | 7 | 5 |  |  | 14 | 7 |  |
| N_1_ | 14 | 16 |  |  | 13 | 21 |  |
| N_2_ | 10 | 19 |  |  | 9 | 11 |  |
| Tumor differentiation |  |  | 0.941 |  |  |  | 0.318 |
| Well/Moderately | 15 | 19 |  |  | 25 | 31 |  |
| Poorly/Undifferentiated | 16 | 21 |  |  | 11 | 8 |  |
| Distance from the anal verge |  |  | 0.893 |  |  |  | 0.509 |
| ≦5cm | 15 | 20 |  |  | 13 | 17 |  |
| > 5cm | 16 | 20 |  |  | 23 | 22 |  |
| Tumor length |  |  | 0.662 |  |  |  | 0.680 |
| ≦4cm | 14 | 16 |  |  | 14 | 17 |  |
| > 4cm | 17 | 24 |  |  | 22 | 22 |  |
| Circumferential extent |  |  | **0.045** |  |  |  | 0.617 |
| ≦50% | 11 | 6 |  |  | 14 | 13 |  |
| > 50% | 20 | 34 |  |  | 22 | 26 |  |
